# Genotype-Phenotype Relationship in Hypertrophic Cardiomyopathy

**DOI:** 10.3390/genes16091090

**Published:** 2025-09-16

**Authors:** Dovilė Žebrauskienė, Eglė Sadauskienė, Roma Puronaitė, Rūta Masiulienė, Ramunė Vaišnorė, Nomeda Bratčikovienė, Nomeda Valevičienė, Jūratė Barysienė, Audronė Jakaitienė, Eglė Preikšaitienė

**Affiliations:** 1 Department of Human and Medical Genetics, Institute of Biomedical Sciences, Faculty of Medicine, Vilnius University, LT-03101 Vilnius, Lithuania; 2 Clinic of Cardiac and Vascular Diseases, Institute of Clinical Medicine, Faculty of Medicine, Vilnius University, LT-03101 Vilnius, Lithuania; 3 Institute of Data Science and Digital Technologies, Faculty of Mathematics and Informatics, Vilnius University, LT-08412 Vilnius, Lithuania; 4 Faculty of Medicine, Vilnius University, LT-03101 Vilnius, Lithuania; 5 Department of Mathematical Statistics, Faculty of Fundamental Sciences, Vilnius Gediminas Technical University, LT-10223 Vilnius, Lithuania; 6 Department of Radiology, Nuclear Medicine and Medical Physics, Institute of Biomedical Sciences, Vilnius University Faculty of Medicine, LT-03101 Vilnius, Lithuania

**Keywords:** hypertrophic cardiomyopathy, genotype, phenotype

## Abstract

Background/Objectives: Hypertrophic cardiomyopathy (HCM) is an inherited disease with genetic and phenotypic variability and an unclear genotype–clinical course relationship. The aim of our study was to assess the phenotypic and molecular characteristics of patients with HCM. Methods: Clinical and genetic data from adult HCM patients treated at a university hospital between 2005 and 2024 were analysed. A comparative analysis of probands with a single pathogenic/likely pathogenic (P/LP) variant and without a P/LP variant was performed. Results: The analysis involved 214 individuals with HCM, 42.1% being females. The median age at HCM diagnosis was 52 (38–62) years. P/LP variants were identified in 92 (43.0%) individuals. Compared to patients without an identified genetic cause, individuals with P/LP variants had a significantly earlier HCM diagnosis (43.5 (32.3–58.0) vs. 54.0 (45.8–65.0) years, *p* < 0.001) and higher maximal thickness on cardiac imaging (17.5 (15.0–21.0) vs. 17.0 (15.0–19.0) mm, *p* = 0.009 on transthoracic echocardiography and 21.0 (18.0–23.0) vs. 18.0 (16.0–20.0) mm, *p* < 0.001 on cardiac magnetic resonance imaging). During the median follow-up of 4.2 (1.6–6.8) years, individuals with P/LP HCM variants had earlier onset of atrial fibrillation (*p* = 0.021), ventricular tachycardia (*p* = 0.004), heart failure composite (*p* = 0.006), and overall composite outcome (*p* = 0.002). No difference between the groups was observed when hazard ratios for clinical outcomes were adjusted by age at HCM diagnosis and gender. Conclusions: Genotype influences HCM phenotype, as patients with P/LP variants experience earlier onset and more pronounced hypertrophy. However, once diagnosed, genotype may not predict the outcomes of HCM.

## 1. Introduction

Hypertrophic cardiomyopathy (HCM) is an inherited, highly heterogeneous disease with variable penetrance and expression. Pathogenic or likely pathogenic (P/LP) variants in genes associated with HCM are identified in 40–60% of HCM patients [1]. Inheritance is predominantly autosomal dominant, with a 50% risk of transmission to offspring. A number of genes have been reported to cause HCM, with eight genes (*MYBPC3*, *MYH7*, *TNNT2*, *TNNI3*, *TPM1*, *ACTC1*, *MYL2* and *MYL3*) demonstrating the strongest supporting evidence [2]. The two most frequently altered genes are *MYBPC3* (40–50%) and *MYH7* (35–40%), coding the myosin-binding protein C and beta-myosin heavy chain, respectively [3]. HCM is a global disease with an estimated prevalence of 1:500 [1,4]. While the phenotypic and clinical characteristics of patients from different races and cultures are thought to be similar, some founder mutations have been identified that are unique to specific ethnic groups [4,5,6].

The exact relationship between the underlying genetic cause and the clinical course remains elusive [3]. Multiple studies have shown that HCM patients with sarcomeric disease-causing DNA variants had earlier presentation of the disease, a family history of HCM, a thicker left ventricle (LV) wall, reverse septal curvature, less significant LV outflow tract (LVOT) obstruction, higher risk of ventricular arrhythmia, and heart failure outcomes [7,8,9,10,11]. Clinical differences between the HCM caused by alterations in different genes are not well-distinguished. Some studies revealed no differences between cardiac imaging parameters (myocardial deformation analysis and cardiac magnetic resonance imaging (CMRi)) in MYBPC3-related and MYH7-related HCM. In contrast, others have reported a higher incidence of atrial fibrillation in patients with pathogenic or likely pathogenic *MYH7* variants [12,13,14]. The aim of our study was to analyse the phenotypic and molecular characteristics of individuals with HCM who were examined and treated at Vilnius University Hospital Santaros Klinikos (VUHSK).

## 2. Materials and Methods

### 2.1. Subsection Study Population and Data Collection

**Figure 1:**
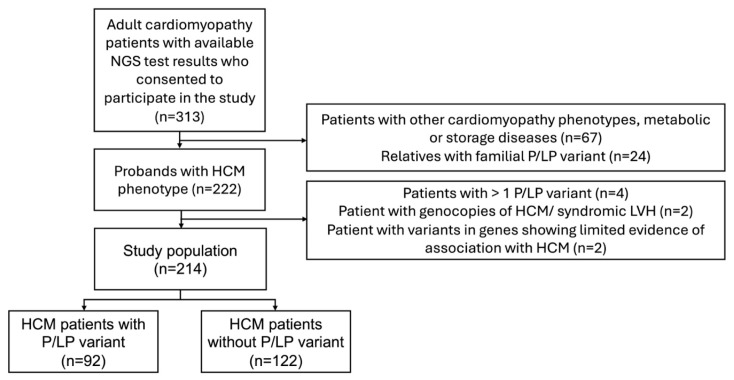
Flowchart of participant selection. HCM—hypertrophic cardiomyopathy; NGS—next-generation sequencing; P/LP—pathogenic/likely pathogenic.

Clinical and genetic data were analysed from unrelated adult (>18 years old) HCM patients treated at VUHSK between 2005 and 2024. The clinical diagnosis of HCM was confirmed by transthoracic echocardiography (TTE) or CMRi with LV wall thickness ≥ 15 mm in any myocardial segment, in the absence of loading conditions, according to the most recent European Society of Cardiology (ESC) guidelines [1]. Only patients who had undergone genetic testing were included in the study. Individuals with more than one pathogenic or likely pathogenic (P/LP) gene variants, variants in genes showing limited evidence of association with HCM, or phenocopies of HCM or syndromic LV hypertrophy (for example, Fabry, amyloidosis, or PRKAG2 cardiomyopathy) were excluded from the final analysis (Figure 1). The study compared data between two groups: individuals with P/LP variants and those without P/LP variants. Clinical manifestation, instrumental examination and next generation sequencing (NGS) data of HCM-related genes were collected. Electrocardiogram (ECG) and TTE were performed on all individuals in the study during primary evaluation. Left ventricular outflow tract obstruction was defined as a peak LVOT gradient ≥ 30 mmHg at rest or during provocation with Valsalva manoeuvre or bicycle stress testing [1]. CMRi with late gadolinium enhancement (LGE) was performed on a subset of patients because some individuals had existing contraindications. HCM sudden cardiac death (SCD) risk at 5 years was estimated using the HCM Risk-SCD tool [1]. Follow-up data included new onset arrhythmias (atrial fibrillation, ventricular tachycardia (VT)), implantation of a pacemaker or implantable cardioverter-defibrillator (ICD), septal reduction therapy (myectomy, alcohol septal ablation), heart transplantation or LV assist device implantation, and mortality. VT was defined as more than three consecutive ventricular beats at a rate > 120 beats/min [1]. 

### 2.2. Study Outcomes

Individual and composite outcomes were defined in the study. Individual outcomes were atrial fibrillation; ventricular tachycardia; sustained advanced heart failure (HF), defined as two consecutive assessments of New York Heart Association (NYHA) functional class III/IV; progression from NYHA functional class I/II to class III/IV; and stroke. The event counts for all-cause mortality, resuscitated cardiac arrest, and appropriate ICD therapy were too low to construct separate composite outcomes. These events were, however, included in the overall composite outcome.

Composite outcomes were characterised as HF and overall composite. Heart failure composite outcomes included first occurrence of cardiac transplantation, LV assist device implantation, LV ejection fraction < 50%, and sustained advanced HF, defined as two consecutive assessments of NYHA class III/IV. Overall composite outcomes involved atrial fibrillation, all-cause mortality, resuscitated cardiac arrest, appropriate ICD therapy, HF composite, and stroke.

### 2.3. Genetic Testing

For the individuals in the study, NGS analysis of blood DNA was performed using the TruSight Cardio Sequencing panel (Illumina Inc., San Diego, CA, USA) in VUHSK from 2015 to 2020 as previously described [15]. Beginning in 2020, NGS of blood DNA was performed using Human Core Exome Kits (Twist Bioscience, South San Francisco, CA, USA) as previously described [16]. The genes associated with HCM were analysed and are listed in Supplementary Material S1. Variants with MAF < 0.02% in control cohorts (ExAC [17], gnomAD [18], and our in-house databases), predicted to be deleterious by prediction tools (SIFT, PolyPhen-2 scores, Mutation Taster), were prioritised. At the time of initial testing, variants were classified according to the guidelines of the American College of Medical Genetics and Genomics (ACMG) [19]. Only variants that passed quality and coverage filters and showed >99.9% detection reliability were analysed. All accessible NGS results of research participants were reannotated to 38 human reference genomes. From August to September 2024, reannotated genomic data were reanalysed and reclassified according to the present guidelines [19]. Copy number variations were reviewed and none were identified.

### 2.4. Statistical Analysis

Continuous variables were summarised using the median and interquartile range (IQR) or means and standard deviation (SD), while categorical variables were presented as numbers and percentages. To compare continuous variables not normally distributed, the Mann–Whitney U for two independent samples, the Wilcoxon signed-rank test for paired samples, and the Kruskal–Wallis test for more than two groups were used. Normally distributed continuous variables were tested using the Welch’s *t*-test for two groups and one-way Analysis of Variance (ANOVA) for more than two groups. Categorical variables were calculated using Pearson’s chi-square or Fisher’s exact tests. All tests were two-sided. A linear mixed-effects model was employed to evaluate the relationship between genotype and maximal LV wall thickness during follow-up.

The Kaplan–Meier analysis was used to determine individual and composite outcomes from birth. Cox proportional hazards models were used to estimate hazard ratios for outcomes between the groups with and without P/LP variants stratified by age at HCM diagnosis and gender.

The difference was considered statistically significant if the *p*-value was less than 0.05. Statistical analysis was performed using SPSS (v. 20) and R (v. 4.4.2) programme packages.

## 3. Results

### 3.1. Demographic and Genetic Characterisation

**Figure 2:**
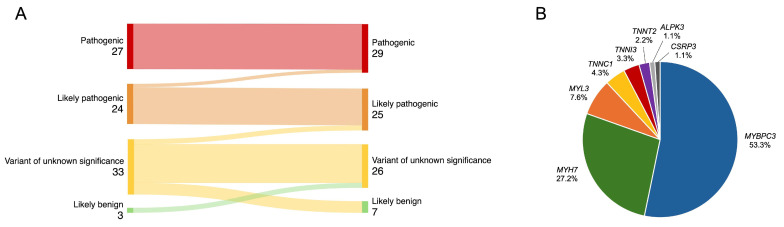
(**A**) Reclassification of variants. (**B**) Distribution of identified P/LP variants in HCM-related genes. HCM—hypertrophic cardiomyopathy; P/LP—pathogenic/likely pathogenic.

Of the 313 patients with cardiomyopathies and available NGS test results, 222 unrelated patients with an HCM phenotype were selected, and the following were excluded from analysis: four individuals with more than one P/LP variant, two with P/LP variants in the *PRKAG2* gene, and two individuals with variants in genes showing limited evidence of association with HCM (*MYH6*, *ANK2*) (Figure 1). The final study analysis involved 214 unrelated HCM patients, 42.1% being females. The median age at HCM diagnosis was 52 (38–62) years (Table 1). The median follow-up was 4.2 (1.6–6.8) years. After re-analysis of reported variants in HCM-related genes, two likely pathogenic variants were upgraded to pathogenic (Figure 2A). From 33 variants of unknown significance (VUS), 7 (21.2%) were downgraded to likely benign, 3 (9.1%) were upgraded to likely pathogenic, and the majority, 23 (69.7%), remained VUS. Three previously unreported variants were reclassified as VUS. According to the results of genetic testing, HCM patients were divided into two groups. The first group involved 92 (43.0%) HCM patients with 48 different P/LP variants, 10 of them novel (Supplemental Material S2, Table S1). Thirty-one individuals had truncating P/LP variants, fifty-nine had missense, and two had in-frame deletions. *MYBPC3* P/LP variants were the most common (*N* = 49), followed by *MYH7* (*N* = 25) and P/LP variants in other genes (*N* = 18) (Figure 2B). The other group involved 122 HCM patients without P/LP variants or with VUS. 

### 3.2. Genotype–Phenotype Relationships

Comparison of the groups of HCM patients with and without P/LP variants revealed that individuals with P/LP variants had a significantly earlier onset of symptoms (41.5 (31.0–56.0) vs. 52.5 (45.0–63.0) years, *p* < 0.001) and HCM diagnosis (43.5 (32.3–58.0) vs. 54.0 (45.8–65.0) years, *p* < 0.001). They also exhibited more pronounced asymmetric septal hypertrophy (Table 1). In contrast, patients without P/LP variants had a more significant comorbidity burden and were less likely to have a family history of HCM or SCD. Patients diagnosed with HCM at an age older than 60 years were more likely to have comorbidities such as primary arterial hypertension (95.2% vs. 59.9%, *p* < 0.001), dyslipidaemia (74.2% vs. 46.7%, *p* < 0.001), coronary artery disease (32.3% vs. 12.5%, *p* < 0.001), and diabetes (17.7% vs. 5.9%. *p* = 0.007) than those diagnosed with HCM before the age of 60. Left ventricular outflow tract obstruction was identified in 37.4% of HCM patients, and an LV apical aneurysm was found in only 4.2%, with no significant difference between the groups. At the primary evaluation, the 5-year HCM SCD risk score was higher in individuals with P/LP variants (2.7 (1.9–3.8) vs. 1.9 (1.5–2.6), *p* **<** 0.001). On ECG, patients with P/LP variants had fewer signs of LV hypertrophy (66.3% vs. 80.3%, *p* = 0.020). They more commonly showed no repolarization abnormalities (46.7% vs. 23.0%, *p* < 0.001), including less negative T waves in the lateral leads (44.6% vs. 73.0%, *p* < 0.001). The presence of a P/LP variant was associated with significantly thicker LV wall (maximal thickness) on cardiac imaging (17.5 (15.0–21.0) vs. 17.0 (15.0–19.0) mm, *p* = 0.009 on TTE and 21.0 (18.0–23.0) vs. 18.0 (16.0–20.0) mm, *p* < 0.001 on CMRi). Subsequently, TTE showed that LV diastolic diameter was smaller in the group of patients with P/LP variants (4.7 ± 0.6 vs. 5.0 ± 0.6 cm, *p* = 0.002). Late gadolinium enhancement was present in most of both groups (88.0% overall), while septal LGE was more common in patients with P/LP variants (59.5% vs. 44.0%, *p* = 0.036). After a median follow-up of 4.2 (1.6–6.8) years, the 5-year HCM SCD risk score remained higher in patients with P/LP variants (2.8 (1.9–4.6) vs. 2.0 (1.5–3.4), *p* < 0.001), reflecting a higher proportion of P/LP-positive individuals meeting the criteria for an ICD implantation (39.1% vs. 18.9%, *p* = 0.001). Only three deaths were reported during the follow-up, all in the group of patients with P/LP variants and not directly related to HCM.

**Figure 3:**
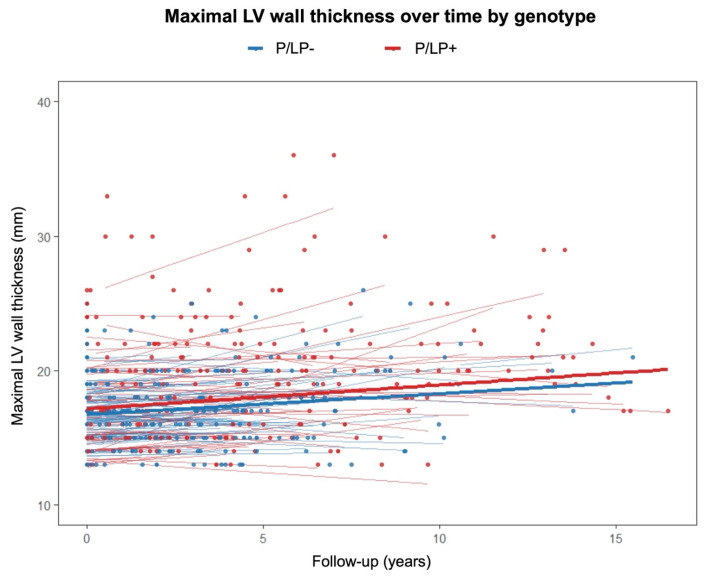
Maximal left ventricular wall thickness on transthoracic echocardiography over time according to genotype. P/LP+—individuals with pathogenic/likely pathogenic variants; P/LP-—individuals without pathogenic/likely pathogenic variants.

A linear mixed-effects model was used to evaluate the relationship between genotype status, follow-up time, and their interaction with regard to maximal LV wall thickness (Figure 3). Hypertrophic cardiomyopathy patients with P/LP variants had a significantly higher baseline LV wall thickness than patients without P/LP variants (*β* = 1.735, 95% confidence interval (CI) [0.859, 2.61], *p* < 0.001). The effect of time alone was not statistically significant (*β* = 0.104, 95% CI [−0.042, 0.249], *p* = 0.162). Similarly, the genotype-by-time interaction (*β* = 0.003, 95% CI [−0.1995, 0.206], *p* = 0.977) did not reach statistical significance.

### 3.3. Clinical Outcomes

**Figure 4:**
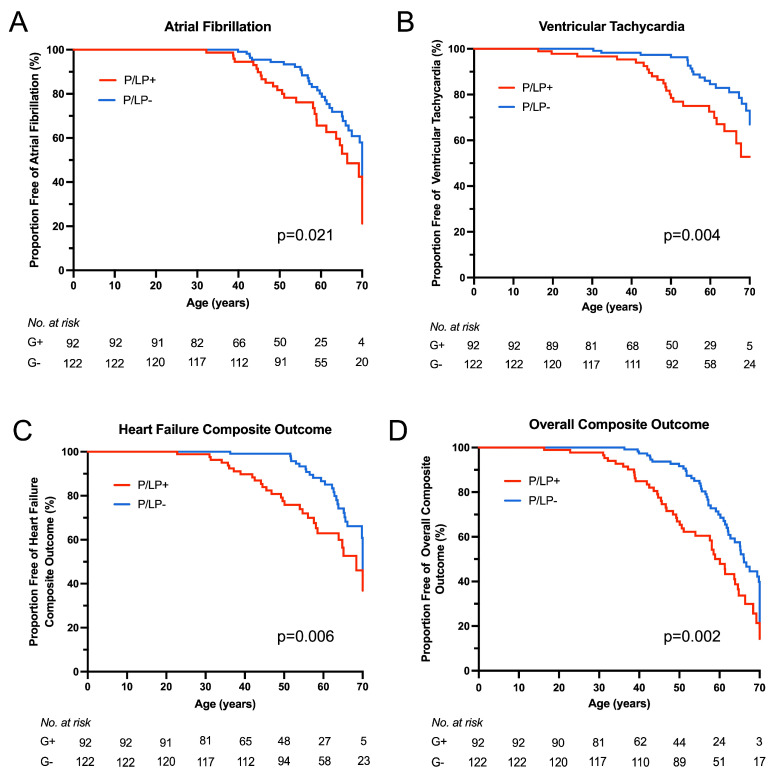
Clinical outcomes according to genotype. Kaplan–Meier survival analysis was performed for clinical outcomes from birth between the groups of patients with known (P/LP+) and unknown (P/LP-) genetic cause of the disease. (**A**) Atrial fibrillation outcome. (**B**) Ventricular tachycardia (non-sustained) outcome. (**C**) Heart failure composite outcome. (**D**) Overall composite outcome.

**Figure 5:**
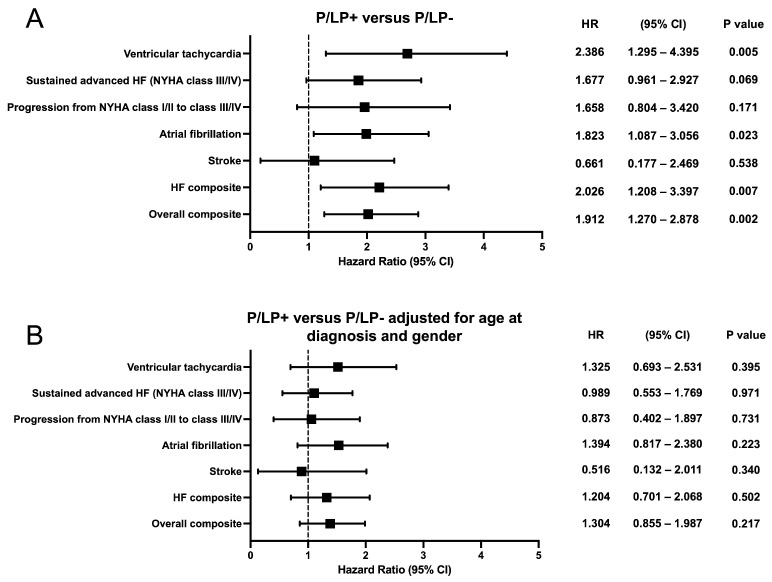
Forest plots showing the hazard ratio and 95% confidence intervals for the individual and composite clinical outcomes according to genotype. (**A**) Hazard ratio between the groups of patients with known (P/LP+) and unknown (P/LP-) genetic cause of the disease. (**B**) Hazard ratio between the groups of patients with known (P/LP+) and unknown (P/LP-) genetic cause of the disease, adjusted for age at diagnosis and gender. CI—confidence interval; HR—hazard ratio.

Kaplan–Meier survival analysis showed earlier onset of atrial fibrillation (*p* = 0.021), ventricular tachycardia (*p* = 0.004), HF composite (*p* = 0.006), and overall composite outcome (*p* = 0.002) for HCM patients with P/LP variants (Figure 4). No statistically significant difference was observed for sustained advanced HF (NYHA functional class III/IV), progression from NYHA functional class I/II to class III/IV, or stroke outcomes between the groups. To compare outcomes between HCM patients with and without P/LP variants, hazard ratios were estimated using Cox proportional hazards regression models (Figure 5). When stratified by age at HCM diagnosis and gender, hazard ratios for neither individual nor composite outcomes differed significantly.

## 4. Discussion

The results of our study showed different clinical and morphological profiles between HCM patients with known and unknown genetic causes of the disease: patients with P/LP variants in HCM-related genes have an earlier onset of HCM, with more pronounced hypertrophy and higher 5-year HCM SCD risk scores, while HCM patients without detected P/LP variants have a higher burden of comorbidities. Comparing outcomes between groups from birth, patients with P/LP variants had earlier onset of arrhythmias (atrial fibrillation and ventricular tachycardia), HF composite, and overall outcomes. When stratifying clinical events by age at HCM diagnosis and gender, we found no significant difference between the groups.

The American College of Cardiology Foundation/American Heart Association (ACC/AHA) guidelines for the management of HCM and the ESC guidelines for the management of cardiomyopathies recommend periodic and systematic reclassification of gene variants every few years [1,20]. Such reclassification can directly affect cascade screening of family members [20]. A single-centre, retrospective variant reclassification according to the current ACMG criteria found that 22% of the HCM-related variants were reclassified [21]. In a recent Spanish HCM subcohort, 16.8% of genetic variants were upgraded and 14.0% were downgraded after re-evaluation [22]. Another study, focusing exclusively on reassessment of VUS, reported that the highest proportion of VUS downgraded, 9.8%, was in patients with HCM, while 1.8% of VUS were upgraded [23]. In our reclassification analysis, 21.2% of VUS were downgraded to likely benign, and 9.1% were upgraded to LP. None of the P/LP variants were downgraded. These results show the importance of regular re-evaluation of gene variants in HCM.

Variants classified as P/LP in HCM-related genes were found in 43.0% of unrelated individuals with HCM. This corresponds with existing evidence that HCM-causing variants are identified in 40–60% of patients [1]. In many studies, HCM individuals with a known genetic cause of the disease tend to have earlier HCM diagnosis than patients without detected P/LP variants [7,8,9,24,25,26]. A similar tendency was observed in our cohort, with about 10 years of HCM diagnosis difference between the groups. Since individuals without P/LP variants are diagnosed with HCM at an older age, they tend to have more comorbidities [7,27].

P/LP variants in HCM-related genes were reported to be associated with a higher maximal LV wall thickness and asymmetric septal hypertrophy [7,9,25,26,28]. However, in a CMRi study, the phenotypic features of MYBPC3-related and MYH7-related HCMs were similar [13]. In our cohort, affected individuals with P/LP variants had a more pronounced asymmetric hypertrophy, with the most remarkable difference on CMRi. Additionally, we performed a linear mixed-effects model to analyse maximal LV wall thickness on TTE during follow-up between the groups of patients with and without P/LP variants. This measurement was chosen because all patients underwent cardiac TTE during primary evaluation, which was repeated during follow-up. Follow-up CMRi was performed on a small proportion of individuals. Patients with P/LP variants had a significantly thicker LV wall at baseline than patients without a known genetic cause of the disease, with no difference in the time effect at follow-up. This means that LV hypertrophy may not progressively thicken in patients with P/LP variants compared to patients with a negative genetic test after HCM diagnosis. These results should be interpreted cautiously, as cardiac imaging tests have high inter-reader variability [29]. There are other causes that may influence HCM phenotype. Intensive exercise may accelerate the progression of HCM due to myocardial damage caused by physical exertion, while moderate-intensity exercise appears to be safe and may improve functional capacity without increasing the risk of arrhythmia [30].

Compared to imaging findings, ECG results were different. Signs of LV hypertrophy on ECG were found in 74.5% of patients. Individuals with P/LP variants had a reduced incidence of signs of LV hypertrophy and negative T waves in the lateral leads. Similar findings were observed in the Portuguese HCM registry, where LV hypertrophy criteria were more pronounced in genotype-negative patients [31]. The authors tried to explain it by less LGE on CMRi and a higher prevalence of hypertension as an environmental modifier, which can lead to higher voltages on ECG in individuals without a known genetic cause of the disease. In another study, the negative T waves in the lateral leads were a significant negative predictor of a positive genotype [28]. These ECG findings correlated with LGE in the corresponding wall on CMRi [32].

Previous studies showed that P/LP variants in sarcomeric genes were associated with increased risk of SCD [9,33]. HCM patients with P/LP variants tend to have a higher median ESC SCD risk score [31] and undergo ICD implantation more often than patients without P/LP variants [5]. The same trends were observed in our study. However, some studies indicate the need to improve the ESC HCM SCD risk scoring tool because it lacks sensitivity [34].

Affected individuals with a known genetic cause of the disease had a median HCM SCD risk score of about 0.8 higher at primary evaluation and during follow-up, and about a 10% higher rate of ICD implantation than HCM patients without P/LP variants. However, the ACC/AHA guidelines for the management of HCM and the ESC guidelines for the management of cardiomyopathies did not include genetic cause as a reliable risk factor for SCD [1,20]. Analysis of the SHaRe registry showed that patients with P/LP variants in sarcomeric genes had earlier onset of clinical outcomes like atrial fibrillation, ventricular arrhythmias, heart failure, and overall composite outcome [8]. In multivariate models adjusted for proband status, gender, and race, the presence of sarcomeric mutation remained an independent predictor of unfavourable events. Further adjustment for age at diagnosis reduced the risk associated with P/LP variants in sarcomeric genes, but the difference remained statistically significant. The development of atrial fibrillation may also be influenced by atrial remodelling, fibrosis, increased atrial size, and impaired LA function [35,36]. Another study reported that clinical outcomes adjusted for age at diagnosis were not significantly different according to genotype [7]. In multivariable models, only age at diagnosis was related to adverse outcomes. Accordingly, the authors concluded that genotype was not a prognostic factor for clinical outcome. Our cohort’s positive genetic test was associated with earlier onset of atrial fibrillation, ventricular tachycardia, HF composite, and overall composite outcome. However, no significant genotype-related difference remained after adjusting hazard ratios for adverse events by age at HCM diagnosis. Thus, after HCM diagnosis, the clinical course was independent of genotype. There is indeed a need for further studies of individual genes and their variants to assess their impact on HCM prognosis. Integration of new technologies such as artificial intelligence into HCM clinical practice can improve patient stratification and management, especially in cases where the genotype alone does not allow for predicting outcomes [37].

### Limitations

Our study had several limitations. First, the retrospective, single-centre study design limited the inclusion of more patients or data. The study cohort was composed of a referral university hospital, which introduced referral bias. Retrospective data collection may have led to a lack of data, unequal follow-up, and variable testing intervals. Cardiac MRI was only performed on a limited number of HCM patients (86%). Others did not undergo the test due to contraindications: iron-based metal implants, cardiac implantable electronic devices, and claustrophobia. The relatively short follow-up period (median 4.2 years) and rare adverse outcomes resulted in all-cause mortality and arrhythmic events (sudden cardiac death, resuscitated cardiac arrest, and appropriate ICD therapy) that could not be analysed separately in the Kaplan–Meier analysis.

## 5. Conclusions

Our study revealed different phenotypic characteristics in HCM patients according to genotype. Individuals with P/LP variants in HCM-related genes tend to have an earlier onset of disease, with more prominent LV hypertrophy and higher 5-year HCM SCD risk. The HCM-related genotype was associated with earlier adverse events, but once HCM had been diagnosed, it was no longer predictive of disease outcomes. These findings may warrant further investigation as to whether genotype-guided HCM management provides additional clinical benefits beyond standard phenotypic assessment.

## Figures and Tables

**Table 1:** Demographics and clinical features in HCM patients with and without P/LP variants.

Variable	All (N = 214)	With P/LP Variants (N = 92)	Without P/LP Variants (N = 122)	*p* Value
Female sex, *n* (%)	90 (42.1%)	38 (41.3%)	52 (42.6%)	0.847
Age at onset of symptoms (years)	50.0 (37.0–61.0)	41.5 (31.0–56.0)	52.5 (45.0–63.0)	**<0.001**
Age at HCM diagnosis (years)	52.0 (38.0–62.0)	43.5 (32.3–58.0)	54.0 (45.8–65.0)	**<0.001**
Septal LV hypertrophy, *n* (%)	180 (84.1%)	85 (92.4%)	95 (77.9%)	**0.004**
Asymmetric LV hypertrophy, *n* (%)	200 (93.5%)	91 (98.9%)	109 (89.3%)	**0.005**
LVOT obstruction, *n* (%)	80 (37.4%)	30 (32.6%)	50 (41.0%)	0.210
LV apical aneurism, *n* (%)	9 (4.2%)	2 (2.2%)	7 (5.7%)	0.306
5-year HCM SCD risk score at primary evaluation	2.2 (1.7–3.2)	2.7 (1.9–3.8)	1.9 (1.5–2.6)	**<0.001**
Family history of HCM, *n* (%)	23 (10.7%)	19 (20.7%)	4 (3.3%)	**<0.001**
Family history of SCD, *n* (%)	29 (13.6%)	18 (19.6%)	11 (9.0%)	**0.026**
Primary arterial hypertension, *n* (%)	150 (70.1%)	51 (55.4%)	99 (81.1%)	**<0.001**
Coronary arterial disease, *n* (%)	39 (18.2%)	8 (8.7%)	31 (25.4%)	**0.002**
Dyslipidaemia, *n* (%)	117 (54.7%)	41 (44.6%)	76 (62.3%)	**0.010**
Diabetes, *n* (%)	20 (9.3%)	4 (4.3%)	16 (13.1%)	**0.029**
NYHA class, *n* (%)				
I	21 (9.8%)	10 (10.9%)	11 (9.0%)	0.903
II	75 (35.0%)	32 (34.8%)	43 (35.2%)	
III	51 (23.8%)	20 (21.7%)	31 (25.4%)	
ECG				
Signs of LV hypertrophy, *n* (%) ^1^	158 (74.5%)	60 (66.7%)	98 (80.3%)	**0.024**
Negative T waves in lateral leads, *n* (%) ^2^	129 (60.6%)	40 (44.0%)	89 (73.0%)	**<0.001**
No repolarization abnormalities, *n* (%) ^2^	71 (33.3%)	43 (47.3%)	28 (23.0%)	**<0.001**
TTE				
Maximal LV wall thickness (mm)	17.0 (15.0–20.0)	17.5 (15.0–21.0)	17.0 (15.0–19.0)	**0.009**
LVdd (cm)	4.8 ± 0.6	4.7 ± 0.6	5.0 ± 0.6	**0.002**
LA size (cm)	4.3 (3.8–4.7)	4.2 (3.7–4.6)	4.3 (4.0–4.8)	0.072
CMRi				
LVEF (%) ^3^	72.0 (63.0–77.0)	71.0 (63.5–76.0)	72.0 (61.5–78.0)	0.679
Maximal LV wall thickness (mm) ^3^	19.0 (17.0–22.0)	21.0 (18.0–23.0)	18.0 (16.0–20.0)	**<0.001**
Myocardial mass index (g/m^2^) ^4^	90 (74.0–104.5)	86.5 (73.0–103.0)	91.0 (75.0–109.0)	0.161
LGE present, *n* (%) ^3^	161 (88.0%)	77 (91.7%)	84 (84.8%)	0.158
LGE present in septum, *n* (%) ^3^	94 (51.1%)	50 (59.5%)	44 (44.0%)	**0.036**
Follow-up and outcomes				
Duration of follow-up (years)	4.2 (1.6–6.8)	4.5 (1.9–8.6)	3.7 (1.1–5.9)	**0.011**
5-year HCM SCD risk score during follow-up	2.3 (1.7–3.6)	2.8 (1.9–4.6)	2.0 (1.5–3.4)	**<0.001**
Septal myectomy, *n* (%)	10 (4.7%)	5 (5.4%)	5 (4.1%)	0.748
Alcohol septal ablation, *n* (%)	17 (7.9%)	6 (6.5%)	11 (9.0%)	0.504
Indication for ICD implantation, *n* (%)	59 (27.6%)	36 (39.1%)	23 (18.9%)	**0.001**
Pacemaker implantation, *n* (%)	13 (6.1%)	3 (3.3%)	10 (8.2%)	0.135
Heart transplantation or LV assisted device implantation, *n* (%)	3 (1.4%)	3 (3.3%)	0 (0.0%)	0.078
Atrial fibrillation, *n* (%)	62 (29.0%)	27 (29.3%)	35 (28.7%)	0.916
Ventricular tachycardia, *n* (%)	43 (20.1%)	23 (25.0%)	20 (16.4%)	0.120
Stroke (%)	12 (5.6%)	3 (3.3%)	9 (7.4%)	0.195
All-cause mortality, *n* (%)	3 (1.4%)	3 (3.3%)	0 (0.0%)	0.078
Sustained advanced heart failure (NYHA functional class III/IV)	53 (24.8%)	22 (23.9%)	31 (25.4%)	0.802
Progression from NYHA functional class I/II to class III/IV	31 (14.5%)	13 (14.1%)	18 (14.8%)	0.898
Heart failure composite	60 (28.0%)	28 (30.4%)	32 (26.2%)	0.498
Overall composite	97 (45.3%)	44 (47.8%)	53 (43.4%)	0.524

^1^*N* = 212; ^2^ *N* = 213; ^3^ *N* = 184; ^4^ *N* = 176. Values are count (%), mean (SD), or median (IQR). CMRi—cardiac magnetic resonance imaging; ECG—electrocardiogram; HCM—hypertrophic cardiomyopathy; ICD—implantable cardioverter-defibrillator; LA—left atrium; LGE—late gadolinium enhancement; LV—left ventricle; LVdd—left ventricular diastolic diameter; LVEF—left ventricular ejection fraction; LVOT—left ventricular outflow tract; NYHA—New York Heart Association heart failure classification; P/LP—pathogenic/likely pathogenic; SCD—sudden cardiac death; TTE—transthoracic echocardiography; Bold formatting is for statistically significant *p* value.

## Data Availability

The main data generated and analysed during this study are included in this article. Any additional information is available from the authors upon request.

## Supplementary Materials

The following supporting information can be downloaded at https://www.mdpi.com/article/10.3390/genes16091090/s1, Supplementary Material S1: The genes, analysed using TruSight Cardio Sequencing panel (Illumina Inc., San Diego, CA, USA); Supplementary Material S2: The list of identified pathogenic and likely pathogenic variants.

## Abbreviations

The following abbreviations are used in this manuscript:

ACC/AHA	American College of Cardiology Foundation/American Heart Association
ACMG	American College of Medical Genetics and Genomics
ANOVA	Analysis of Variance
CI	Confidence interval
CMRi	Cardiac magnetic resonance imaging
ECG	Electrocardiogram
ESC	European Society of Cardiology
HCM	Hypertrophic cardiomyopathy
HF	Heart failure
ICD	Implantable cardioverter-defibrillator
IQR	Interquartile range
LA	Left atrium
LGE	Late gadolinium enhancement
LV	Left ventricle
LVdd	Left ventricular diastolic diameter
LVEF	Left ventricular ejection fraction
LVOT	Left ventricular outflow tract
NGS	Next generation sequencing
NYHA	New York Heart Association
P/LP	Pathogenic/likely pathogenic
SCD	Sudden cardiac death
SD	Standard deviation
TTE	Transthoracic echocardiography
VT	Ventricular tachycardia
VUHSK	Vilnius University Hospital Santaros Klinikos
