# Supplementary material for: Genotype-Phenotype Relationship in Hypertrophic Cardiomyopathy
Source: Genes (Basel). 2025 Sep 16;16(9):1090. doi: 10.3390/genes16091090 (PMC12470014; doi:10.3390/genes16091090)
Supplement: Supplementary file 1 [file genes-16-01090-s001.zip › genes-3830911-supplementary/Supplemental material S2.pdf]

Table S1. The list of identified pathogenic and likely pathogenic variants.

Gene	Classification	Protein-coding RNA reference sequence	Nucleotide change	Protein (amino acid) reference sequence	Amino acid change	Mutation type	Novel variant*	No. of patients with variant
ALPK3	LP	NM_020778.5	c.2956del	NP_065829.4	p.Ala986GlnfsTer12	Frameshift	Yes	1
CSRP3	P	NM_003476.5	c.120C>A	NP_003467.1	p.Cys40Ter	Nonsense	Yes	1
MYBPC3	P	NM_000256.3	c.772G>A	NP_000247.2	p.Glu258Lys	Missense	No	1
MYBPC3	P	NM_000256.3	c.1457G>A	NP_000247.2	p.Trp486Ter	Nonsense	No	1
MYBPC3	P	NM_000256.3	c.1483C>T	NP_000247.2	p.Arg495Trp	Missense	No	2
MYBPC3	P	NM_000256.3	c.1505G>A	NP_000247.2	p.Arg502Gln	Missense	No	7
MYBPC3	LP	NM_000256.3	c.1543_1545del	NP_000247.2	p.Asn515del	Inframe deletion	No	1
MYBPC3	P	NM_000256.3	c.1996A>T	NP_000247.2	p.Lys666Ter	Nonsense	Yes	2
MYBPC3	P	NM_000256.3	c.2603_2612del	NP_000247.2	p.Gly868AlafsTer8	Frameshift	Yes	1
MYBPC3	P	NM_000256.3	c.2610del	NP_000247.2	p.Ser871AlafsTer8	Frameshift	No	1
MYBPC3	P	NM_000256.3	c.3190+5G>A	NP_000247.2	p.?	Splaising	No	2
MYBPC3	LP	NM_000256.3	c.3407_3409delACT	NP_000247.2	p.Tyr1136del	Inframe deletion	No	1
MYBPC3	P	NM_000256.3	c.3467dup	NP_000247.2	p.Pro1157AlafsTer12	Frameshift	No	6
MYBPC3	P	NM_000256.3	c.3642G>A	NP_000247.2	p.Trp1214Ter	Nonsense	No	1
MYBPC3	LP	NM_000256.3	c.3683_3684dup	NP_000247.2	p.Met1229AlafsTer9	Frameshift	No	1
MYBPC3	P	NM_000256.3	c.3697C>T	NP_000247.2	p.Gln1233Ter	Nonsense	No	12
MYBPC3	LP	NM_000256.3	c.3763G>A	NP_000247.2	p.Ala1255Thr	Missense	No	8
MYBPC3	P	NM_000256.3	c.3773T>G	NP_000247.2	p.Leu1258Ter	Nonsense	No	1
MYBPC3	P	NM_000256.3	c.3815-1G>A	NP_000247.2	p.?	Splaising	No	1
MYH7	P	NM_000257.4	c.746G>A	NP_000248.2	p.Arg249Gln	Missense	No	1
MYH7	LP	NM_000257.4	c.936C>A	NP_000248.2	p.Phe312Leu	Missense	Yes	1
MYH7	LP	NM_000257.4	c.947G>A	NP_000248.2	p.Gly316Glu	Missense	Yes	1
MYH7	LP	NM_000257.4	c.958G>A	NP_000248.2	p.Val320Met	Missense	No	2
MYH7	LP	NM_000257.4	c.1231G>A	NP_000248.2	p.Val411Ile	Missense	No	1
MYH7	LP	NM_000257.4	c.1314G>C	NP_000248.2	p.Trp438Cys	Missense	No	1
MYH7	P	NM_000257.4	c.1357C>T	NP_000248.2	p.Arg453Cys	Missense	No	1
MYH7	LP	NM_000257.4	c.1385T>C	NP_000248.2	p.Ile462Thr	Missense	Yes	1

MYH7	LP	NM_000257.4	c.1711G>A	NP_000248.2	p.Gly571Arg	Missense	No	1
MYH7	P	NM_000257.4	c.1988G>A	NP_000248.2	p.Arg663His	Missense	No	2
MYH7	P	NM_000257.4	c.2011C>T	NP_000248.2	p.Arg671Cys	Missense	No	1
MYH7	LP	NM_000257.4	c.2129C>T	NP_000248.2	p.Pro710Leu	Missense	No	1
MYH7	LP	NM_000257.4	c.2185G>C	NP_000248.2	p.Ala729Pro	Missense	No	1
MYH7	P	NM_000257.4	c.2207T>C	NP_000248.2	p.Ile736Thr	Missense	No	1
MYH7	LP	NM_000257.4	c.2390C>T	NP_000248.2	p.Ala797Val	Missense	Yes	1
MYH7	LP	NM_000257.4	c.2492A>C	NP_000248.2	p.Lys831Thr	Missense	Yes	1
MYH7	P	NM_000257.4	c.2608C>T	NP_000248.2	p.Arg870Cys	Missense	No	1
MYH7	P	NM_000257.4	c.2609G>A	NP_000248.2	p.Arg870His	Missense	No	2
MYH7	LP	NM_000257.4	c.4259G>A	NP_000248.2	p.Arg1420Gln	Missense	No	2
MYH7	P	NM_000257.4	c.5134C>T	NP_000248.2	p.Arg1712Trp	Missense	No	1
MYH7	P	NM_000257.2	c.5135G>A	NP_000248.2	p.Arg1712Gln	Missense	No	1
MYL3	LP	NM_000258.3	c.170C>G	NP_000249.1	p.Ala57Gly	Missense	No	3
MYL3	LP	NM_000258.3	c.382G>T	NP_000249.1	p.Gly128Cys	Missense	No	3
MYL3	LP	NM_000258.3	c.433A>C	NP_000249.1	p.Asn145His	Missense	Yes	1
TNNC1	LP	NM_003280.3	c.430A>G	NP_003271.1	p.Asn144Asp	Missense	No	4
TNNI3	P	NM_000363.5	c.433C>G	NP_000354.4	p.Arg145Gly	Missense	No	1
TNNI3	P	NM_000363.5	c.434G>A	NP_000354.4	p.Arg145Gln	Missense	No	2
TNNT2	P	NM_001276345.2	c.304C>T	NP_001263274.1	p.Arg102Trp	Missense	No	1
TNNT2	LP	NM_001276345.2	c.886C>T	NP_001263274.1	p.Arg296Cys	Missense	No	1

*Variants checked on 25/10/2024.

LP - likely pathogenic; P – pathogenic.
